# Laparoscopic Retrieval of a Peritoneal Mouse

**DOI:** 10.1155/2010/624825

**Published:** 2010-09-15

**Authors:** Dara O. Kavanagh, Diarmaid Moran, Robert Flynn, Paul C. Neary

**Affiliations:** Division of Colorectal Surgery, Departments of Colorectal Surgery and Urology, Adelaide and Meath Incorporating the National Children's Hospital, Tallaght, Dublin 24, Ireland

## Abstract

A 67-year-old Caucasian male was referred by the urology service with a history of incomplete bowel emptying. He complained of tenesmus. MRI scan suggested a leiomyoma lying anterior to the rectum. He underwent examination under anaesthesia and attempted endorectal ultrasound and biopsy. However, the lesion seemed to migrate cranially and was impalpable. At laparoscopy, a mobile, unattached, 5.5 × 5 × 3.5, cream-coloured ‘egg was retrieved from the retrovesical space. Histology confirmed a hyalinised fibrocollagenous lesion lined with mesothelium. A comprehensive review of the literature is presented.

## 1. Introduction

Peritoneal loose bodies or “peritoneal mice” are asymptomatic incidentalomas predominantly found at abdominal surgery or autopsy [1]. They usually are from 0.5 to 2.5 cms in diameter. Giant peritoneal mice (>5 cms) can be associated with symptomatology due to a mass effect. They are thought to evolve from torsion and separation of the appendices epiploicae. Others have suggested that large loose bodies can be formed by the accumulation of peritoneal serum in the appendices epiploicae [2]. Herein, we discuss a giant peritoneal mouse masquerading as a pelvic tumour causing alarming rectal symptoms.

## 2. Case Report

**Figure 1:**
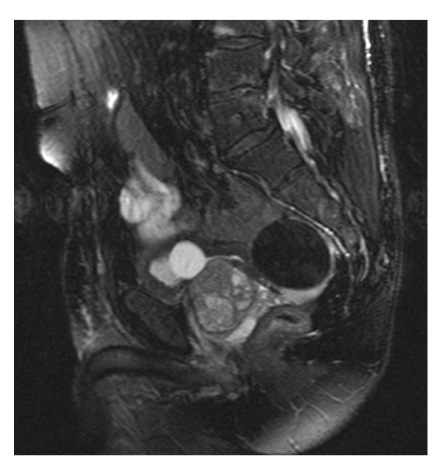
T2-weighted (fat-suppressed) sagittal image of the pelvis reveals a 5.5 cm retrovesical lesion lying anterior to the rectum. It is well circumscribed. There is no pelvic sidewall lymphadenopathy.

**Figure 2:**
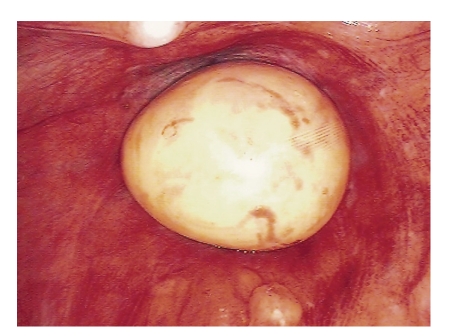
A cream-coloured ovoid mass is seen anterior to the rectum with the tip of the urethral catheter visible anterior to it.

A 67-year-old Caucasian male was referred from the urology service following a previous laparoscopic nephrectomy for clear-cell renal carcinoma. He complained of progressive narrowing of his stool with associated symptoms of tenesmus. He was intermittently distended. He denied rectal bleeding or weight loss. His past medical history was unremarkable apart from essential hypertension controlled with an ACE inhibitor. Digital rectal examination was poorly tolerated and subsequent flexible endoscopy suggested an extra rectal lesion encroaching on the posterior wall. Computed tomography (CT) and magnetic resonance imaging (MRI) showed a smooth-surfaced, well-circumscribed egg-shaped lesion measuring 5.5 × 5.3 × 4.4 cms with central calcifications posterior to the bladder but anterior and separate from the rectum (Figure 1). Examination under anaesthesia and attempted ultrasound-guided biopsy of this very symptomatic lesion were unsuccessful. The lesion became impalpable and was no longer visible on endorectal ultrasonography. Endoscopy was unremarkable. After the informed consent was obtained, a diagnostic laparoscopy was performed. This revealed a mobile cream-coloured egg anterior to the rectum which was removed via a 4-cm infraumbilical incision (Figure 2). Histology revealed a mesothelium-lined nodule of extensively hyalinised fibrocollagenous tissue with central calcification consistent with a “peritoneal loose body”. He made a favourable recovery and was discharged home the following day. He remained symptom-free.

## 3. Discussion

It is generally agreed that peritoneal loose bodies arise from appendices epiploicae. These fat-filled visceral peritoneal pouches exist along the antimesenteric taenia of the entire colon [3]. Their physiological significance is unclear. When they are subjected to torsion and subsequent infarction, inflammatory changes related to peritoneal adhesions, volvulus, bowel obstruction, and perforation may follow. They can mimic acute appendicitis among other causes of acute abdominal pain. In cases of chronic torsion, ischemia ensues leading to saponification, calcification of the fat contents, and atrophy of the pedicle. The appendix epiploica detaches from the colon and becomes a peritoneal loose body or “peritoneal mouse”. It is not uncommon to find small loose bodies during laparotomy [4]. Normally, they have little or no pathological significance, and their prevalence is not clearly documented. However, giant loose bodies (>5 cms) are very rare, and the process of their development has not yet been fully elucidated [5]. Recent reports have identified a peritoneal loose body in the presence of a unilateral absence of adnexal structures, thereby postulating childhood adnexal torsion with autoamputation and gradual calcification [1].

The current case describes a giant peritoneal loose body case masquerading as a pelvis lesion causing significant rectal symptomatology related to extrinsic compression. A pelvic MRI depicted the lesion with features consistent with leiomyoma. There was low signal on the T1- and T2-weighted images with central low signal consistent with central calcification. It is important to differentiate leiomyomas from other lesions such as fibromata, desmoid tumors, teratomas, metastatic lesions of ovarian cancer, calcification of lymph nodes, and mesenteric cysts [6]. All of these lesions tend to enhance with contrast except peritoneal loose bodies. It was critical to further evaluate the current case with endorectal ultrasonography, palpation, and biopsy to outrule a malignant process in view of the progressive nature of the symptoms. This evaluation resulted in a cranial migration of the lesion such that it remained impalpable and not amenable to ultrasonographic visualisation. Endoscopic examination of the rectum revealed normal rectal mucosa.

After the informed consent was obtained, the patient underwent laparoscopic evaluation. This revealed a mobile cream-coloured egg lying anterior to the rectum. This was removed. The rest of the peritoneal contents were normal. Microscopy of the lesion revealed hyalinised fibrocartilaginous tissue lined with mesothelial tissue. This lamellar pattern of fibrous tissue with a scarcity of cellular tissue is typical of what is seen histologically in a peritoneal body [7].

## 4. Conclusion

Giant peritoneal loose bodies are rare clinical entities. Imaging is often inconclusive when symptomatic laparoscopic retrieval is recommended to alleviate symptoms and outrule malignancy.

##  Consent

Written informed consent was obtained from the patient for publication of this case report and accompanying images. A copy of the written consent is available for review by the Editor-in-Chief of this journal.

##  Competing Interests 

The authors declare that they have no competing interests.

##  Authors' Contributions

Dara O. Kavanagh performed the surgery mentioned in the paper. DC collected the images and made a comprehensive literature review. RF proofread the paper. PC contributed significantly to the writing of the paper. All authors read and approved the final paper.
